# An Observation of Coordinated Collaboration in Wild Brown Skua (*Stercorarius antarcticus*)

**DOI:** 10.1002/ece3.72670

**Published:** 2025-12-12

**Authors:** George J. F. Swan, Guillam E. McIvor, Dominic L. Cram

**Affiliations:** ^1^ Instituto de Conservación, Biodiversidad y Territorio, Facultad de Ciencias Forestales y Recursos Naturales Universidad Austral de Chile Valdivia Chile; ^2^ HX Expeditions London UK; ^3^ Department of Behavioural and Cognitive Biology University of Vienna Vienna Austria; ^4^ Centre for Ecology, Evolution and Conservation, School of Biological Sciences University of East Anglia Norwich UK

**Keywords:** avian cognition, collaboration, cooperation, loose‐string paradigm, skua, social cognition

## Abstract

Examples of non‐human animals cooperatively problem‐solving during foraging are rare in the natural world. However, early reports on *Stercorariidae* species describe mutualistic collaboration in which pairs of birds apparently coordinate to remove meat from animal carcasses. For the first time, we provide video evidence of this behaviour in brown skua (*Stercorarius antarcticus*) on the Antarctic Peninsula and analyse it against four categories of collaboration described in the literature. We observe that this feeding behaviour does have characteristics of some of the dependent measures required for actively coordinated collaboration. Skua were coordinating their actions over space and time, including appearing to wait for partners when working together and using a different feeding technique when the partner was unavailable. Skua held the carcass without shaking when waiting for the collaborator, and then repeatedly pulled in unison. By contrast, they shook or pecked the carcass when alone. Experimental studies are now needed to determine the extent to which skua understand the causal role of their partner. The behaviour we analyse is noteworthy, not only for its apparent rarity in wild animals, but because it provides a real‐world example of the task captive animals are trained to undertake in classic cooperation experiments (coordinated pulling to obtain a food reward). We highlight *Stercorariidae* species as promising models for future research on avian social cognition and identify potential research questions that would help uncover both the ultimate and proximate mechanisms driving their collaborative behaviour.

## Graphical abstract

We present the first video evidence of cooperative food processing in wild brown skuas ( *Stercorarius antarcticus* ) on the Antarctic Peninsula. Our observations show that pairs coordinated their actions over space and time, consistent with actively coordinated collaboration. Our findings bridge experimental research on animal cooperation with natural foraging behaviour, highlighting skuas as valuable models for investigating the cognitive and evolutionary bases of collaboration.

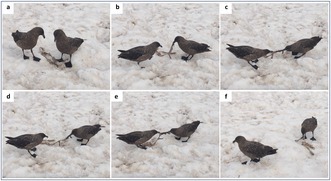

## 1 Introduction

Collaborative behaviours, in which individuals actively coordinate their actions to achieve shared benefits, represent one of the most cognitively demanding forms of cooperation (Duguid and Melis 2020). While many cooperative interactions arise when individuals gain benefits unobtainable alone (Brosnan and de Waal 2002), collaboration requires that partners adjust their actions in relation to one another. Such behaviours range from simpler, conditional responses to complex coordination that is likely to be underpinned by more advanced cognitive processes (Duguid and Melis 2020). Interest in collaboration stems from its potential to illuminate the evolution of cognition, social behaviour, and mechanisms of social interaction (Trivers 1971). Although primates have been the main focus for studies of animal collaboration (Kappeler and van Schaik 2006), evidence increasingly shows that similar capacities have evolved independently in birds (Heaney et al. 2017). Collaborative behaviour therefore offers an important comparative perspective for understanding how complex cognition evolves.

In the wild, pairs or groups of birds are known to actively coordinate their behaviour in a number of contexts. Common examples include cooperative breeding (Cockburn 2006), predator mobbing (Carlson and Griesser 2022), and vocal duets (Hall 2009). Birds can also coordinate during foraging at spatial–temporal scales (Baldan and van Loon 2022), or in the time that they return to the nest (Shen et al. 2010). However, examples of wild birds collaborating to acquire or process food are rare, largely restricted to reports of cooperative hunting in raptors and corvids (Ellis et al. 1993; Yosef and Yosef 2010). Despite these observations of avian collaborative foraging under natural conditions, detailed studies exploring the motivations, actions, and coordination required to achieve mutual benefit are sparse.

In experimental settings, research on collaboration in birds has focused on *psittacine* (parrot) and *corvidae* (crow) species with apparatuses that provide a food reward when birds work together. First conducted by Crawford (1937), the ‘Loose String Paradigm’, in which both subjects must pull a string simultaneously to get a reward, has become the “benchmark test” for examining whether animals intentionally coordinate to cooperate (Heaney et al. 2017). Although both parrots and crows can achieve this task (Heaney et al. 2017; Massen et al. 2015; Péron et al. 2011; Seed et al. 2008; Tassin de Montaigu et al. 2020), success is not sufficient to demonstrate ‘true collaboration’ as joint pulling can be a consequence of independent but simultaneous actions (Melis and Semmann 2010). For interactions to qualify as truly collaborative, at least one of the participants must behave in a way that improves coordination between the two, demonstrating knowledge that the partner's actions causally contribute to their own success (Noë 2006). To test this, experimenters have delayed the arrival of the partner to the task and observed whether the subject waited to pull the string. These studies have shown that while kea parrots ( *Nestor notabilis* ) (Heaney et al. 2017) and ravens ( *Corvus corax* ) (Asakawa‐Haas et al. 2016; but see Massen et al. 2015) were able to wait for a partner, other corvid (Seed et al. 2008) and parrot (Péron et al. 2011; Tassin de Montaigu et al. 2020; Torres Ortiz et al. 2020) species generally fail to do so and are therefore probably achieving success in the task without taking their partners' role into account. Broadly, progress has been hampered by a narrow taxonomic scope and difficulty interpreting the actions of animals performed under artificial conditions. Observations from groups other than corvids and parrots, under natural conditions, are thus required.

Bird species in the family *Stercorariidae*, known as skuas (also ‘jaegers’ in North American English), may offer an under‐exploited opportunity to study cognition. These are predatory seabirds with traits commonly associated with enhanced cognition (Ducatez et al. 2015; Emery et al. 2007; Sol et al. 2016), including long‐term pair bonds, slow development, generalist diets, and flexible foraging strategies (Andersson 1971; Carneiro et al. 2015; Votier et al. 2007; Young 1963). They also form complex social networks with fission–fusion dynamics and forage opportunistically in flocks (Carneiro et al. 2015; Madani 2020).

Examples of coordinated cooperation in skuas are promising, but anecdotal. Schulz (2004) described the coordinated distraction of adult penguins to depredate eggs. Other reports mention ‘cooperative feeding’ in most skua species, including great ( *Stercorarius skua* ), brown ( *Stercorarius antarcticus* ), south polar ( *Stercorarius maccormicki* ), long‐tailed ( *Stercorarius longicaudus* ), and pomarine ( *Stercorarius pomarinus* ) (Andersson 1971; Furness 1987; Maher 1970; Stonehouse 1956; Young 1963). Unlike birds of prey, skuas do not use their feet to hold food items while they feed; instead, carcass parts are broken down by holding with the beak and shaking (Andersson 1971; Stonehouse 1956). However, during cooperative feeding, pairs were observed pulling carcasses from different sides, reducing handling time and increasing food gained (Andersson 1971; Maher 1970). Such accounts are suggestive of collaboration but could also reflect identical, independent actions, as commonly seen in tug‐of‐war food competition, or in captive birds that can solve cooperative string‐pulling tasks by immediately pulling any available string (e.g., Tassin de Montaigu et al. 2020). However, some cooperative feeding observations include details that are suggestive of more active coordination, including birds soliciting partners for the task, taking complementary roles, and equally sharing benefits (Stonehouse 1956; Young 1963; G. Miller, personal communication, 03 November 2025). Despite further anecdotal reports (Madani 2020; Schulz 2004; Travers 2021), these behaviours are yet to be appraised in light of our most up‐to‐date criteria and understanding of cooperative behaviour (Duguid and Melis 2020; Melis and Raihani 2023). Here, we report observations of potential coordinated collaboration between two brown skuas as they feed on a penguin carcass. We describe and critically evaluate the observation in the light of published categories of animal collaboration.

## 2 Methods and Materials

Within the literature, there are different definitions regarding the spectrum of cooperative behaviour (e.g., Bailey et al. 2013; Noë 2006). However, as a framework by which to analyse skua behaviour, we use the four types of collaborative behaviour defined by Duguid and Melis (2020). We chose this framework as it provides detailed definitions of each category, including possible dependent measures. These are summarised in Table 1.

**TABLE 1:** Definitions of the different levels of collaboration summarized from Duguid and Melis (2020).

Category	Summary
By‐product collaboration	Joint action arising from individual decision‐making based on external events, not on conspecifics' actions
Socially influenced collaboration	Individuals are more likely to pursue a particular behaviour (such as hunting) in the presence of others but do not intentionally facilitate coordination
Actively coordinated collaboration	Individuals intentionally coordinate with others and have knowledge that the presence and actions of the partner causally contribute to their own success
Collaboration based on shared intentionality	Individuals coordinate to achieve tasks, each with knowledge of shared goals and partner intentions

## 3 Observation

**FIGURE 1:**
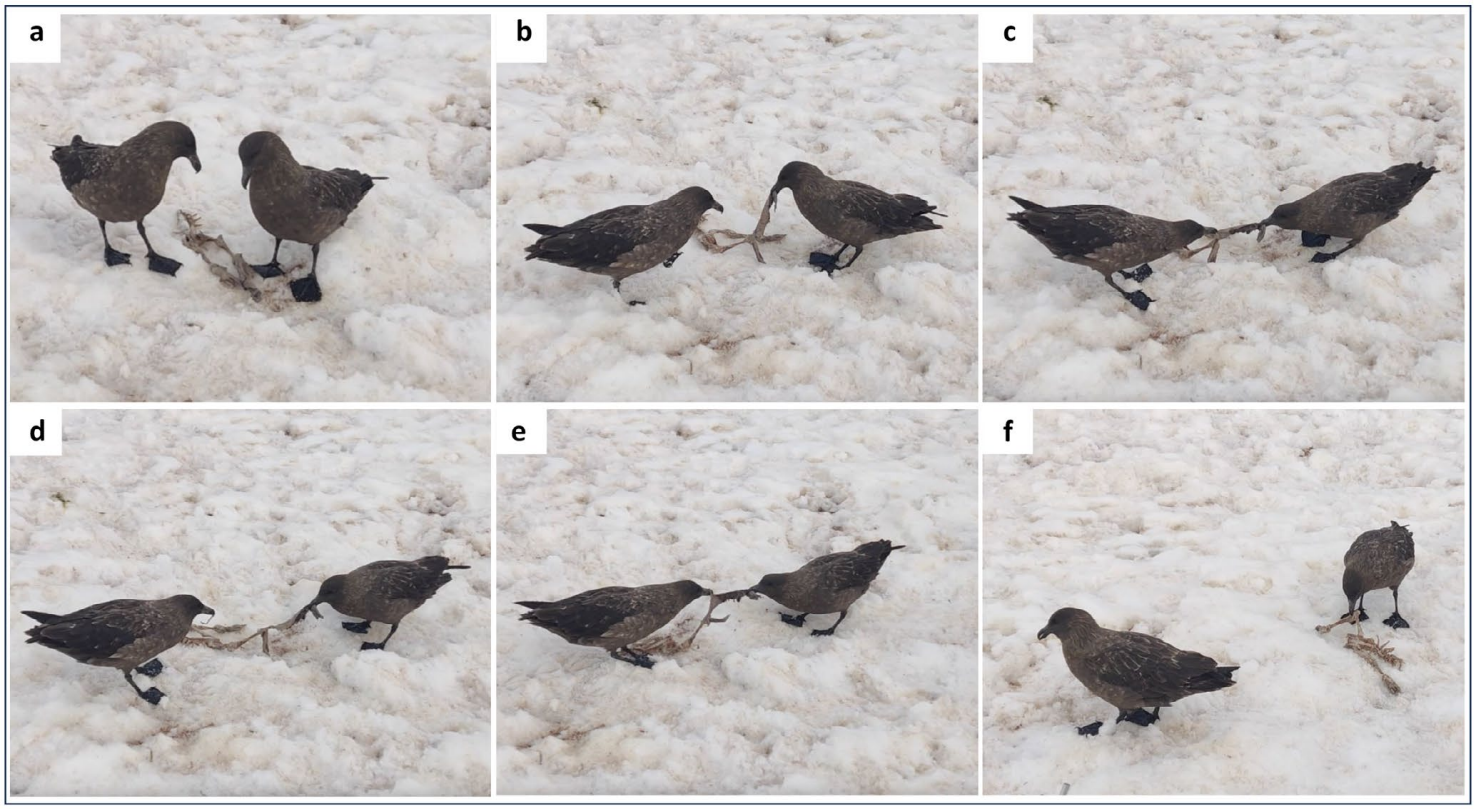
Stills from video footage (Data S1) showing two subantarctic brown skua cooperating to pull meat off a gentoo penguin carcass. (a) Bird A (*left*) and Bird B (*right*) approach part of the carcass together. (b) Bird B holds the carcass up. (c) Both pull in unison. (d) Bird A tears off a piece of skin and eats it while Bird B continues to hold. (e) Bird A returns, and they pull in unison. (f) Bird B feeds by shaking the carcass when Bird A is no longer engaged.

On the 25th of November 2024 on Cuverville Island, Antarctic Peninsula, a single subantarctic brown skua (*S. a. lonnbergi*) was observed feeding on skeletal carcass sections of a gentoo penguin ( *Pygoscelis papua* ). After several minutes, this bird began calling, and a second skua landed within a meter. The two then went through a territorial display, with both birds calling and posturing together, indicating a shared pair‐bond (Stonehouse 1956). Shortly after, both birds approached part of the penguin carcass, and one bird picked it up in its beak, taking no action until the other bird grasped a dangling piece. Both birds then pulled in unison, most often in a regular rhythm, until one ripped a small piece off, the carcass broke apart, or one bird lost its grip (a sequence hereafter referred to as a ‘bout’) (Figure 1). Though it is not easy to tell whether birds were pulling in unison or one bird was holding the carcass while the other pulled (causing the holding bird's body to move as it held firm), it should be noted that across bouts, both birds succeeded in detaching an edible piece, suggesting either mutual tugging or turn‐taking of which bird anchored and which pulled. When a bout finished, the bird still holding the carcass did not let go, nor did it turn or retreat to monopolise the food. Instead, it held the large carcass piece until its partner could resume tugging in synchrony again. Indeed, in several instances, the bird left holding the piece walked towards its partner, rather than away. This continued until the carcass section split into two similarly sized parts that were then swallowed by the birds (Figure 1). Having consumed these parts, they moved onto a different section of the carcass and started again. At no point did either skua try to shield the carcass they were carrying or act aggressively towards the other bird, though we twice observed individuals try, unsuccessfully, to steal a detached scrap from the other bird—after which they returned to the cooperative feeding behaviour.

Foraging in this manner continued for ca. 15 min. During this, we recorded a 4:18 min video with a Xiaomi smartphone camera (Data S1). Analysis of the video footage at 0.5× playback speed allowed us to quantify aspects of cooperative investment and reward (Data S2). The video shows 32 bouts of joint feeding. Counting the number of synchronised tugs per bout revealed a mean of 2.8 Hz (standard deviation (SD) = 1.02) or 168.2 per minute. Each bout lasted an average of 3.59 s (range = 1–39, SD = 6.68). The average time that one bird held the carcass before the other started pulling was 1.5 s (range = 0–7, SD = 1.46). Although skua often shook the carcass to dislodge scraps of meat when foraging alone (personal observation, GJFS), the holder only shook the carcass during 2 of the 32 waiting periods before the partner engaged. At least one bird achieved a food reward during 27 (84.4%) of the bouts, but both birds were rewarded only once (3.1%). Of these, Bird A obtained 16 small carcass pieces (smaller than beak size) and 1 large piece (larger than beak size) while Bird B obtained 8 small carcass pieces and 2 large pieces. Bird B had the longest number of bouts without reward (10). After the joint feeding behaviour ended, when Bird A disengaged from the interaction and turned the other way, the skuas were observed trying to feed on parts of the carcass on their own. This solo feeding involved pecking or shaking the carcass (e.g., Data S1, 04:14). Although the first skua called at the beginning of the feeding event, and both called together shortly after the second's arrival, no calls were heard during the joint feeding period. It was not possible to discern the age (though neither were juveniles) or sex of either bird.

## 4 Discussion

The behaviour described in this study has the hallmarks of collaboration with the shared goal of facilitating food processing and consumption. Although this is a single observation and studies of this nature present difficulties in drawing firm conclusions regarding the cognitive mechanisms involved in cooperation, video evidence provides detail to support this claim, namely over the 32 recorded bouts: both birds (1) face the partner with the carcass section rather than attempting to guard it; (2) display a level of inhibitory control by waiting with the carcass without shaking until their partner engaged, albeit for a short period; (3) repeatedly pull in unison; (4) hold the carcass in the same position while their partner consumes a scrap of meat, awaiting the resumption of cooperation; and (5) move together to various carcass sections to continue collaborative food processing. When taken together, the evidence supports that one or both birds in the pair are observing and adapting to their partner's behaviour over space and time, thus satisfying the minimal requirements for ‘actively coordinated collaboration’ (Table 1, Duguid and Melis 2020). We suggest, therefore, that our footage confirms the collaborative feeding behaviour reported across skua species by early studies (Andersson 1971; Furness 1987; Maher 1970; Stonehouse 1956; Young 1963). Below, we discuss the potential cognitive mechanisms underpinning this behaviour, the ecological significance of synchronised pulling behaviour, and the potential comparative value of our observations. We also detail how future studies could deepen our understanding of this behaviour.

Our observations raise questions about how collaborative feeding in brown skua may have evolved. While behaviourally complex complementary roles have been reported in skua during cooperative hunting (Schulz 2004), the carcass‐pulling behaviour we describe here could arise either from complex cognition or from simpler conditional rules. In other taxa, associative learning and straightforward heuristics can lead to behaviours that superficially resemble collaboration (Bailey et al. 2013), making it difficult to infer underlying mechanisms from field observations alone (Duguid and Melis 2020). Several evolutionary routes remain plausible. Collaborative feeding may have originated as a self‐serving strategy during complex group hunting, with the benefits of enhanced coordination selecting for more sophisticated cognitive skills (Melis and Raihani 2023). Alternatively, it may reflect simple heritable conditional rules, such as “pull when partner holds,” that require little cognitive complexity but nonetheless yield cooperative outcomes (Noë 2006). Regardless of its proximate basis, this feeding technique apparently increases both the speed and amount of food obtained for long‐tailed skua (Andersson 1971). Although further data are required to confirm this for brown skua, such benefits could provide a clear fitness advantage. Thus, collaborative feeding in skuas is likely to have been favoured by selection, whether supported by advanced cognition or by simpler behavioural strategies. The presence of this behaviour on the Antarctic Peninsula would illustrate how cooperative strategies may underlie the capacity of birds to colonise extreme environments (Cornwallis et al. 2017).

Although both birds obtained multiple food items during carcass processing, we did not observe evidence of payoff symmetry (a characteristic of mutualistic interactions in which both partners gain near‐immediate benefits; Mayerhoff and Brosnan 2022). Instead, rewards were typically asymmetric, with only one bird benefiting from each pulling bout. This delay in benefits, sometimes extending to ten bouts without reward for a given individual, suggests that skuas may alternately hold less profitable or inedible parts of the carcass to facilitate their partner's access to food (as implied by Young 1963). Such prosocial behaviour could be mediated either by positive affective evaluations of a partner (emotional bookkeeping) or by memory of specific past interactions (precise bookkeeping) (Raihani and Bshary 2011). Though emotional bookkeeping is a less cognitively demanding mechanism for maintaining cooperation, it typically depends on stable, long‐term social bonds. However, both early studies and anecdotal observations indicate that cooperative food processing in skuas can occur not only between breeding pairs but also among non‐breeding individuals (Stonehouse 1956; Young 1963; R.W. Furness, pers. comm., 25 September 2025). Whether these individuals were close kin is not clear. For precise bookkeeping, such as direct reciprocity, there is a scarcity of evidence in non‐primates, something attributed to its cognitive requirements for partner recognition and memory of partner‐specific past interactions (Melis and Raihani 2023). Yet, experimental research has shown that some birds have such cognitive capacities (Müller et al. 2017) and it is feasible that brown skua will be able to recognise conspecifics outside of their pair‐bond and recall previous co‐foraging outcomes, given that they are able to recognise individual humans and remember interactions with them (Lee et al. 2016). Careful experimentation is required to test direct reciprocity by clarifying whether skuas can anticipate delayed benefits, rather than acting solely for immediate gain (Melis and Raihani 2023; see Box 1).

BOX 1 **We suggest six potential research questions that would help uncover the prevalence of coordinated collaboration and both the ultimate and proximate mechanisms driving it.**

1. *Is coordinated collaboration widespread in brown skua and other carrion‐feeding species?* Studies of different colonies and skua species could record the prevalence of collaboration and both the social context and food resources that produce it. For example, collaborative food processing by both brown and south polar skua on the Palmer Archipelago, Antarctica, is apparently highest in the weeks after the Adélie ( *Pygoscelis adeliae* ) and gentoo penguins hatch (B. Houston, pers. comm., 18 March 2025). Colonies with marked birds would also reveal which birds are choosing to work together. Observing collaboration between birds without clear social relationships would suggest the behaviour it is not maintained by emotional bookkeeping. More broadly, scrutiny of tug‐of‐war interactions in other predatory or scavenging species may uncover similar instances of collaboration in other taxa.
2. *How does coordinated collaboration develop in brown skuas?* This question would address whether the behaviour is instinctive even in naïve individuals that have never witnessed or taken part before, or whether it must be learnt either through personal or social learning. The answer will improve our understanding of the behaviours' inheritance, which may rely on unbroken chains of peer observation.
3. *What cognitive mechanisms are required for actively coordinated collaboration in brown skuas?* The proximate mechanisms behind collaboration can be explored by observing behaviour during variations of the loose strong paradigm. For example, knowledge, or lack thereof, of the causal role of the partner has been inferred from subjects delaying actions (Heaney et al. 2017), recruiting partners (Melis et al. 2006) or choosing the appropriate test (Seed et al. 2008) when faced with the loose‐string paradigm. Addressing this question will reveal whether simple rule‐learning, or more complex cognitive processes, are required. Testing subjects in a variety of contexts would provide the most compelling evidence (Duguid and Melis 2020). Further comparative studies across species could reveal whether similar cognitive mechanisms underlie cooperative behaviours in diverse ecological contexts and selection pressures or reveal the circumstances that led to it evolving in some lineages and not others.
4. *How is coordinated synchrony achieved?* The absence of audible calls during the foraging observation and the ability of skua to follow behavioural clues from humans when foraging (Danel et al. 2023) suggests that skua use visual coordination to collaborate. This could be tested with the addition of an opaque barrier to the loose‐string paradigm to disrupt visual contact between individuals. Cooperation in capuchin monkeys ( *Cebus apella* ) stopped when such a barrier was introduced suggesting that individuals were coordinating actions using visual, not auditory, signals (Mendres and de Waal 2000). Video analysis of head movements, or head‐mounted accelerometer data, would also reveal whether there are consistent rhythms or rates of pulling, or whether pull synchrony is negotiated and improved during a trial.
5. *Are the benefits underpinned by mutualism or direct reciprocity?* This could be tested experimentally with two apparatuses; one that provides food rewards to both sides, and another that only provides the reward to one side when pulled simultaneously. Repeated use, and how a pair divides access to the side with the reward, would illuminate if helping a partner was merely a by‐product of individual objectives (mutualism) or of a more advanced cognitive ability to anticipate downstream benefits (direct reciprocity).
6. *Does collaboration affect partner choice?* The apparent benefits of cooperative feeding for pairs opens the intriguing possibility that cooperative (and noncooperative) interactions influence mate choice. Cooperation in birds appears increase with the quality of the relationship between individuals, possibly due to increased tolerance for close proximity (Asakawa‐Haas et al. 2016). However, little is known about how animals keep track of cooperative interactions (Kings et al. 2023) or whether more cooperative individuals are better quality mates (Covas and Doutrelant 2019) and further study of skua would present an opportunity to explore such questions. Long‐term studies showing survival and divorce rates in relation to success at collaborative cooperation could reveal the fitness consequences of effective cooperation between mates.

Experimental research exploring avian cooperation is most often conducted in captivity, leading to questions about the ecological validity and evolutionary significance of performance in behavioural assays (Noë 2006; Pritchard et al. 2016). Our observations are relevant to these concerns in two ways. First, they demonstrate real‐world validity of the loose‐string paradigm, as successful foraging in this species and context relies on the same specific performance required to resolve this widely used cognitive assay. Second, a reliance on captive assays of cognition and cooperation is partly due to the time required for subjects to become familiar with and approach the experimental apparatus (Heaney et al. 2017, but see Jacobs and Osvath 2015 for evidence of string‐pulling in wild birds) with neophobia being a major impediment to performing such experiments in many suitable species (Miller et al. 2022). We therefore highlight brown skuas as a tractable species for such studies, because they readily approach human‐made objects and are highly motivated by food rewards (Danel et al. 2023; Danel et al. 2021). Moreover, the ecological relevance of synchronised collaborative pulling makes them ideal models to explore the ‘loose string paradigm’ on birds in the wild for the first time, by addressing key unanswered questions (Box 1).

Our observation adds valuable taxonomic diversity to comparative attempts to identify the life‐history correlates of advanced cognitive abilities in birds. Skuas show many life‐history traits in common with other birds capable of complex cognition. They exhibit social complexity and fission–fusion dynamics (Amici et al. 2008), long‐term pair bonds (Emery et al. 2007), extended juvenile periods, slow pace‐of‐life, and long lifespans (Sol et al. 2016), dietary generalism (Ducatez et al. 2015), and flexible foraging strategies including kleptoparasitism (Carneiro et al. 2015; Morand‐Ferron et al. 2007; Votier et al. 2007). Large relative brain sizes are often correlated with enhanced cognitive skills (but see Logan et al. 2018), and while to our knowledge such data are not available for brown skua, the endocranial volume or brain mass of close relatives is substantially *smaller* than expected for their body size ( *Stercorarius longicaudus* , Smith and Clarke 2012; *Stercorarius maccormicki*, Jiménez‐Ortega et al. 2020). Our findings therefore support the view that the evolution of cognitive complexity is better understood in relation to life‐history traits than to brain size alone (Sol et al. 2016).

## 5 Conclusion

This study presents the first formal analysis of cooperative food processing in wild skua, placing the behaviour within the framework of actively coordinated collaboration as defined by Duguid and Melis (2020). The evidence of spatial and temporal symmetry, inhibitory control, sustained joint pulling, and tolerance at close range suggests that skua coordinate their actions to improve foraging success. This finding bridges a gap between controlled cooperation experiments and natural foraging behaviours, highlighting the ecological relevance of such cognitive skills.

Given their flexible social systems, diverse foraging strategies, and apparent willingness to engage in coordinated tasks, skua species are promising candidates for experimental work on cooperation in the wild. Comparative and manipulative studies across skua species could reveal whether their collaboration is driven by mutualism, reciprocity, or simpler associative rules, offering insight into the evolutionary pathways of complex social cognition. Recognising and studying these behaviours in the field not only deepens our understanding of seabird ecology but also expands the taxonomic scope of animal cooperation and cognition research.

## Author Contributions

**George J. F. Swan:** conceptualization (lead), data curation (lead), formal analysis (lead), investigation (lead), methodology (equal), resources (equal), software (lead), supervision (equal), visualization (lead), writing – original draft (lead), writing – review and editing (equal). **Guillam E. McIvor:** conceptualization (equal), formal analysis (supporting), investigation (supporting), methodology (supporting), supervision (equal), writing – review and editing (supporting). **Dominic L. Cram:** conceptualization (equal), data curation (supporting), formal analysis (supporting), funding acquisition (lead), investigation (supporting), methodology (equal), resources (supporting), software (supporting), supervision (equal), visualization (supporting), writing – original draft (supporting), writing – review and editing (lead).

## Funding

G.E.M. was supported by a Vienna Science and Technology Fund (WWTF) [10.47379/VRG21011].

## Conflicts of Interest

The authors declare no conflicts of interest.

## Supporting information


**Data S1:** ece372670‐sup‐0001‐DataS1.mov.

**Data S2:** ece372670‐sup‐0002‐DataS2.xlsx.

## Data Availability

All relevant data is within the paper and its Supporting Information—S1 and S2files.
